# Variability in pathogenicity prediction programs: impact on clinical diagnostics

**DOI:** 10.1002/mgg3.116

**Published:** 2014-12-03

**Authors:** Lauren C Walters-Sen, Sayaka Hashimoto, Devon Lamb Thrush, Shalini Reshmi, Julie M Gastier-Foster, Caroline Astbury, Robert E Pyatt

**Affiliations:** 1 Center for Human Genetics, Inc.Cambridge, Massachusetts; 2 Department of Pathology and Laboratory Medicine, Nationwide Children's HospitalColumbus, Ohio; 3 Department of Pediatrics, The Ohio State University College of MedicineColumbus, Ohio; 4 Department of Pathology, The Ohio State University College of MedicineColumbus, Ohio

**Keywords:** Diagnostics, pathogenicity, prediction, sequencing, variants

## Abstract

Current practice by clinical diagnostic laboratories is to utilize online prediction programs to help determine the significance of novel variants in a given gene sequence. However, these programs vary widely in their methods and ability to correctly predict the pathogenicity of a given sequence change. The performance of 17 publicly available pathogenicity prediction programs was assayed using a dataset consisting of 122 credibly pathogenic and benign variants in genes associated with the RASopathy family of disorders and limb-girdle muscular dystrophy. Performance metrics were compared between the programs to determine the most accurate program for loss-of-function and gain-of-function mechanisms. No one program correctly predicted the pathogenicity of all variants analyzed. A major hindrance to the analysis was the lack of output from a significant portion of the programs. The best performer was MutPred, which had a weighted accuracy of 82.6% in the full dataset. Surprisingly, combining the results of the top three programs did not increase the ability to predict pathogenicity over the top performer alone. As the increasing number of sequence changes in larger datasets will require interpretation, the current study demonstrates that extreme caution must be taken when reporting pathogenicity based on statistical online protein prediction programs in the absence of functional studies.

## Introduction

Clinical diagnostic laboratories are often faced with the challenge of describing novel sequence variants in disease-associated genes. While the establishment of mutation databases has helped to catalog previously reported variants, the interpretation of a truly novel unpublished variant still remains challenging. As whole-exome sequencing (WES) expands into clinical practice, there is the potential for the identification of a vast number of unique sequence variants that will need to be analyzed for potential impact on the specific phenotype of the individual.

The gold standard for variant analysis is functional experimentation in an appropriate system. However, this is beyond the scope of most clinical laboratories. Instead, laboratories frequently utilize online pathogenicity prediction programs, along with traditional literature and database inquiries. These programs attempt to assign a pathogenicity rating for missense variants using different components concerning sequence evolution and protein structure. As each program utilizes a different algorithm, the interpretations can be significantly different for a given variant. In addition, while guidelines for the use of prediction tools for research applications have been published, no consensus rules for their clinical validation and application exist (Vihinen 2013). Consequently, identification of the best possible pathogenicity prediction program for an individual laboratory's needs is of paramount importance.

We evaluated the performance of 17 publicly available pathogenicity prediction programs. As input, we generated a dataset of credibly pathogenic and benign variants for two distinct disease mechanisms: gain-of-function/autosomal dominant mutations in the RASopathy family of disorders and loss-of-function/autosomal recessive mutations in the limb-girdle muscular dystrophy (LGMD) family of disorders. With an eye toward clinical implications, this study sought to identify the most effective pathogenicity prediction program or set of programs for the clinical laboratory diagnostician.

## Materials and Methods

### Variant selection

As no clinically-validated benchmark datasets are available, pathogenic and benign variants were selected for analysis based on a review of clinical sequencing results from the Molecular Genetics Laboratory at Nationwide Children's Hospital (NCH) from 2006 to 2013. Additional benign variants were identified through an Ensembl search for missense variants in the appropriate gene transcript. Only variants resulting in missense changes were used in these analyses.

### Variant classification

**Figure 1:**
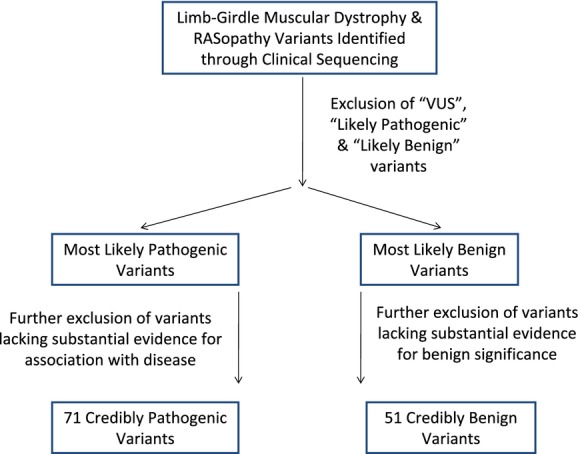
Scheme for the selection of variants used in this study. Functional studies are the gold standard by which to establish the disease association (pathogenic) or normal variation (benign) status of any sequence variant. Unfortunately, functional studies have only been preformed for a small portion of identified variants. To ensure the greatest likelihood that we were using pathogenic and benign variants to examine this series of prediction programs, we used this selection criteria to establish our dataset. Thirty-six additional benign variants were extracted from Ensembl as few had been identified by clinical sequencing analysis. For detailed descriptions of criteria, please see the Materials and Methods section.

An ideal dataset for this exercise would consist of variants which had all undergone functional analysis to concretely establish their pathogenic link to a particular disease phenotype. As there is not a substantial number of variants in the RASopathy- or LGMD-associated genes that meet this criterion, we instead adopted an approach to select variants with the greatest overall likelihood of being pathogenic or benign (Fig.1). Variants defined as credibly pathogenic for the purposes of this study met all of the following criteria: published in a peer-reviewed journal as disease-associated OR listed as such in a curated database (HGMD, Leiden Muscular Dystrophy); variant identified through clinical testing in a patient with clinical diagnosis OR in a sample used in validation studies; and sequenced in the NCH Molecular Genetics Laboratory. Validation samples were specimens from verified cases obtained from other diagnostic laboratories. Any amino acid changes identified internally as *likely* pathogenic due to a lack of published verification of pathogenicity were excluded. These included variants that resulted in a different amino acid change than that which had previously been reported as pathogenic for a given codon (i.e., *PTPN11* p.Asp106Gly rather than p.Asp106Ala).

Variants defined as credibly benign met all of the following criteria: seen in at least two sources that utilized large populations (1000 Genomes, Exome Sequencing Project, etc.); entry in dbSNP OR 1000 Genomes databases with a minor allele frequency listed as greater than 0.001 (for RASopathy genes) OR 0.010 (for LGMD genes) in at least one population; and listed as “validated” in dbSNP OR published in a peer-reviewed journal as benign. Of note, a recent analysis of pathogenic variants found in the ESP dataset did not list any variants in any of the genes in our dataset (Dorschner et al. 2013).

### Variant analysis

All variants were analyzed using the following publicly available prediction programs: PolyPhen-2 (Adzhubei et al. 2010), SIFT (Kumar et al. 2009), PMut (Ferrer-Costa et al. 2004), SNPs3D (Yue et al. 2006), PANTHER (Thomas et al. 2003), FATHMM (Shihab et al. 2013), MutationTaster (Schwarz et al. 2010), Condel (Gonzalez-Perez and Lopez-Bigas 2011), PROVEAN (Choi et al. 2012), Mutation Assessor (Reva et al. 2011), MutPred (Li et al. 2009), nsSNPAnalyzer (Bao et al. 2005), PhD-SNP (Capriotti et al. 2006), SNAP (Bromberg and Rost 2007), and SNPs&GO (Calabrese et al. 2009). The majority of these programs had only a single algorithm option for analysis, with two exceptions. For PolyPhen-2, this included HumDiv and HumVar algorithms (Adzhubei et al. 2010); for FATHMM, this included Weighted and Unweighted algorithms (Shihab et al. 2013).

Programs were used in the manner of a basic user without high-level bioinformatics skills. Default settings were used for each program, with the exception of PhD-SNP; for this program, the option of a 20-fold cross-validation prediction was used. For programs utilizing a multiple sequence alignment, the native alignment was used. Detailed descriptions of each program are listed in Data S1.

### Program performance

The performance of each of the protein prediction programs was analyzed by comparing a variety of statistical measures using the following calculations: 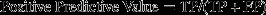


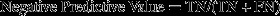


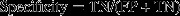


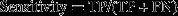


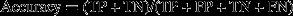


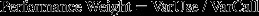


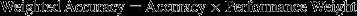


For these calculations, TP, True Positives, pathogenic variants called as pathogenic; FP, False Positives, benign variants called as pathogenic; TN, True Negatives, benign variants called as benign; and FN, False Negatives, pathogenic variants called as benign. For Performance Weight, VarUse, number of variants that had usable pathogenicity calls, that is, Damaging or Benign [possible predictions were not included (i.e., Possibly Damaging), nor were predictions with low reliability]; and VarCall, number of variants that generated output predictions from a given program.

## Results

### Study design

Two distinct datasets populated by variants defined as credibly pathogenic or benign were used as input for 17 different pathogenicity prediction programs (Thomas et al. 2003; Ferrer-Costa et al. 2004; Bao et al. 2005; Capriotti et al. 2006; Yue et al. 2006; Bromberg and Rost 2007; Calabrese et al. 2009; Kumar et al. 2009; Li et al. 2009; Adzhubei et al. 2010; Schwarz et al. 2010; Gonzalez-Perez and Lopez-Bigas 2011; Reva et al. 2011; Choi et al. 2012; Shihab et al. 2013). The first dataset consisted of 35 credibly pathogenic and 19 credibly benign variants in genes involved in RASopathy syndromes (Table S1). The proteins implicated in this family of disorders all interact via the RAS/ERK/MAPK signaling pathway. Mutations in any of the involved genes result in increased pathway signaling, which causes increased cell proliferation and abnormal responses to growth factor, hormones, cytokines, and cell adhesion molecules. All RASopathy mutations are inherited in an autosomal dominant manner, and those analyzed in this study operate under a gain-of-function mechanism. This dataset included three *PTPN11* mutations that have been seen in both individuals with Noonan syndrome and those with LEOPARD syndrome.

The second dataset consisted of 36 credibly pathogenic and 32 credibly benign variants in genes involved in LGMD (Table S2). The proteins implicated in this family of disorders comprise different components of the dystrophin–glycoprotein complex in muscle (*SGCA*, *SGCB*, *SGCG*, *SGCD*), the dysferlin complex and its interacting proteins in the sarcolemma (*DYSF*, *CAPN3*), or other muscle-related functions (*ANO5*, *FKRP*). The presence of two mutations (in *trans*) in these genes results in a lack of functional protein, thereby disrupting the balance of muscle function. The LGMD mutations analyzed in this study are inherited in an autosomal recessive manner and operate under a loss-of-function mechanism.

Pathogenicity prediction program output was either word-based (pathological, benign, etc.) or numerical (output score). For word-based outputs (PolyPhen-2, SIFT, PMut, FATHMM, MutationTaster, Condel, nsSNPAnalyzer, PhD-SNP, SNAP, and SNPs&GO), the exact output was recorded as stated. For numerical outputs (SNPs3D, PANTHER, PROVEAN, Mutation Assessor, and MutPred), the output was transformed as described in Data S1. For consistency across programs, Probably Damaging was considered to be synonymous with Damaging, Pathological, Deleterious, Disease-Causing, Disease, or Non-Neutral; Possibly Damaging was considered to be synonymous with Possibly Deleterious; and Benign was considered to be synonymous with Tolerated, Non-Deleterious, Polymorphism, or Neutral. Output was listed as N/A if no output was produced for a given variant.

### Program performance

The lack of output due to the inability to predict pathogenicity for a specific variant was a noticeable problem with several of the programs. In particular, programs that relied on premade Hidden Markov Models (PANTHER, FATHMM-Weighted), published protein structures (nsSNPAnalyzer), or the use of specific isoforms (SNPs3D, Mutation Assessor, SNPs&GO) did not produce predictions for a number of variants. The program most significantly affected in this manner was nsSNPAnalyzer, as it required a protein structure in the ASTRAL database for the basis of its pathogenicity predictions; only 61 (50.0%) of 122 variants yielded a prediction. For the RASopathy genes, no protein structures were available for *RAF1*, *MAP2K2*, or *SHOC2* (9 total variants). For the LGMD genes, no protein structures were available for *SGCA*, *SGCB*, *SGCD*, *SGCG*, *ANO5*, *FKRP*, or *DYSF* (49 total variants).

Our goal was to query these programs with variants demonstrating the strongest evidence for pathogenic or benign impact. As such, for programs with reliability score outputs, all outputs were recorded but only those above a given reliability threshold (5 on a 0–9 scale) were used in statistical analyses (see Data S1). Programs that generated reliability scores included PMut, MutationTaster, PhD-SNP, SNAP, and SNPs&GO. The weakest performers were PMut and SNAP, with only 69 (56.6%) of 122 variants with data receiving reliable pathogenicity predictions. The LGMD dataset was affected slightly more than the RASopathy dataset in both cases. For PMut, only 36 (52.9%) of 68 LGMD variants had reliable calls, as opposed to 33 (61.1%) of 54 RASopathy variants. Similar results were seen for SNAP; only 50.0% of LGMD variants (34/68) and 64.8% of RASopathy variants (35/54) had reliable calls.

Similarly, for programs with nondichotomous outputs (i.e., including “possible” pathogenicity), all outputs were recorded to differentiate these programs from those which completely failed to produce a prediction; however, only clearly damaging or clearly benign outputs were used in statistical analyses. This was done for consistency between dichotomous and nondichotomous outputs. Programs affected by this analysis step included those which self-assigned “possible” pathogenicity (PolyPhen-2, SIFT) and those with numerical outputs which were assigned “possible” pathogenicity in this analysis (SNPs3D, PANTHER, PROVEAN, Mutation Assessor, and MutPred). The biggest drop-off in analyzable data was seen for Mutation Assessor; of 100 variants with predictions, only 66 (66.0%) were clearly pathogenic or clearly benign. As seen with the reliability programs, the LGMD dataset was affected more than the RASopathy dataset. Only 27 (58.7%) of 46 LGMD variants with predictions were clearly called, as opposed to 39 (72.2%) of 54 RASopathy variants.

**Figure 2:**
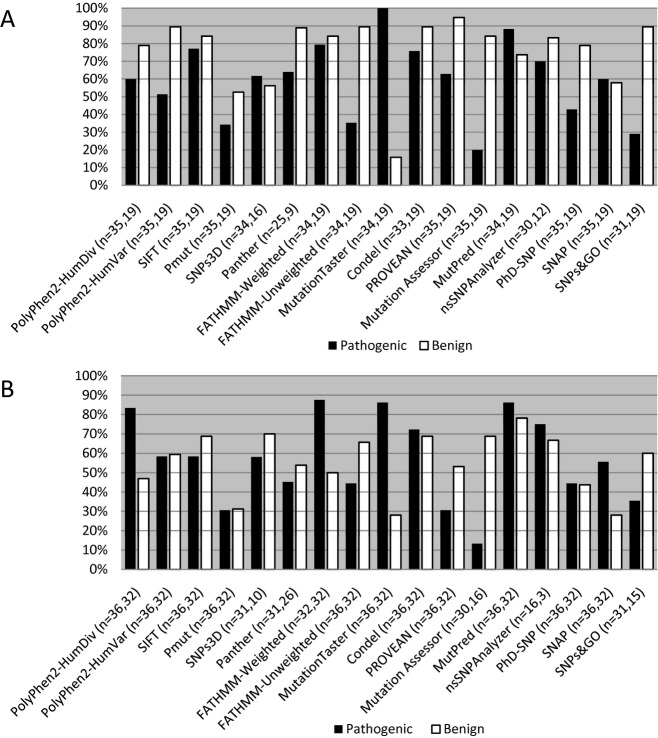
Percentage of correct predictions. The ability of the prediction programs to correctly assign either pathogenic (black) or benign (white) status to variants in the RASopathy dataset (A) and the LGMD dataset (B) is shown. The program used and the number of variants with prediction outputs (pathogenic, benign) are listed below the graph. Percentages were generated by dividing the number of variants predicted correctly by the number of variants with prediction outputs for each class (pathogenic or benign). The RASopathy dataset contained 35 credibly pathogenic variants and 19 credibly benign variants. The LGMD dataset contained 36 credibly pathogenic variants and 32 credibly benign variants.

The ability of individual programs to correctly predict the pathogenicity of a variant ran the gamut from highly accurate to very poor performance. The percentage of correct predictions for both credibly pathogenic (true positive) and credibly benign (true negative) variants in the RASopathy/gain-of-function dataset is shown in Figure2A. Interestingly, the prediction capabilities of certain programs varied between the two types of variants. MutationTaster was able to correctly predict 100.0% of credibly pathogenic mutations but only 15.8% of the credibly benign variants; these values amounted to the strongest and weakest performance, respectively. Other standout programs were PROVEAN, with the best performance with credibly benign variants (94.7% correctly predicted), and Mutation Assessor, with the weakest performance with credibly pathogenic mutations (20.0% correctly predicted). Representative outputs for a selection of credibly pathogenic RASopathy variants can be seen in Table S3A.

A similar range of performance capabilities was observed for the LGMD/loss-of-function variants (Fig.2B). FATHMM (weighted option) was able to correctly predict 87.5% of the credibly pathogenic mutations, while Mutation Assessor could only predict 13.3% of the same mutations. With the credibly benign variants, the best performance was observed with MutPred (78.1% correctly predicted). The weakest was seen with MutationTaster and SNAP, with only 28.1% of credibly benign variants correctly predicted. In general, overall performance was less than the RASopathy dataset, perhaps due to the inherent uncertainty when assessing a single loss-of-function variant without the recessive partner seen in the disease state. Representative outputs for a selection of credibly pathogenic LGMD variants can be seen in Table S3B.

Very few variants in our dataset received correct predictions from all programs tested in this analysis. If nonreliable outputs (those with reliability scores below the cutoff discussed in the Data S1 section) were disregarded, six credibly pathogenic variants and six credibly benign variants were correctly predicted by all programs (Table S4). Therefore, only 12 (9.8%) of 122 variants were reliably classified as benign or pathogenic by all programs. The distribution of correct calls was split relatively evenly between the two datasets. Five RASopathy variants (two credibly pathogenic and three credibly benign) were completely correct (5/54 = 9.3%), as opposed to seven (four credibly pathogenic and three credibly benign) LGMD variants (7/68 = 10.3%).

### Analysis of program performance measures

Several statistical measures were used to assess the performance of each individual pathogenicity prediction programs. These included positive predictive value (PPV), negative predictive value (NPV), specificity, sensitivity, accuracy, performance weight, and weighted accuracy. Performance weight, the proportion of variants with useable predictions, was used to normalize the accuracy values between those programs with low amounts of useable data and those with high amounts. All measures were calculated for the individual datasets and the combined dataset.

**Figure 3:**
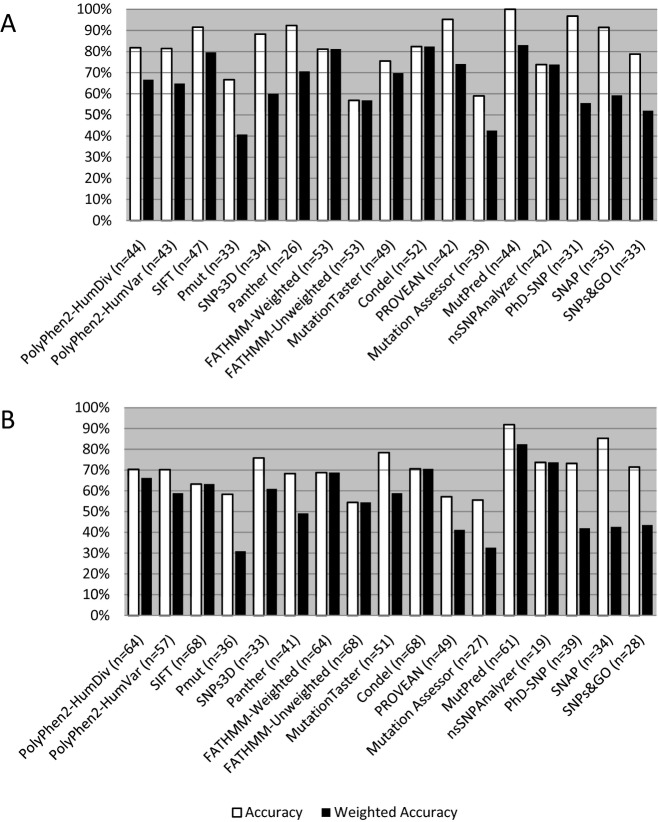
Accuracy of prediction programs in the RASopathy and LGMD datasets. Both accuracy (white) and weighted accuracy (black) are shown for the prediction programs analyzed. The number of variants with usable prediction calls are listed for each individual program. (A) Gain-of-function RASopathy variants (*n* = 54); (B) Loss-of-function LGMD variants (*n* = 68).

Statistical measures for the RASopathy/gain-of-function dataset can be seen in Table1, with accuracy values shown in Figure3A. The best overall performer in this dataset was MutPred, with a weighted accuracy of 83.0%. This value also represented the unweighted accuracy of this program, as MutPred was able to produce usable data for all variants with predictions. Perfect specificity and PPV scores were achieved with several programs, highlighting the ability of these programs to correctly assign pathogenic status to dominant, gain-of-function mutations. However, only two programs, MutationTaster and MutPred, had perfect sensitivity and NPV scores. The weakest overall performer with this dataset was PMut, with a weighted accuracy of 40.7%. MutationTaster had both the lowest specificity (20.0%) and the lowest PPV (73.9%). Mutation Assessor had the lowest sensitivity (30.4%), while FATHMM-Unweighted had the lowest NPV (45.9%).

**Table 1:** Statistical measures for program performance in the RASopathy dataset

Program	PPV^1^	NPV^1^	Specificity^1^	Sensitivity^1^	Accuracy^1^	PWeight^1^	WAccuracy^1^
PolyPhen2-HumDiv	0.955	0.682	0.938	0.750	0.818	0.815	0.667
PolyPhen2-HumVar	1.000	0.680	1.000	0.692	0.814	0.796	0.648
SIFT	0.931	0.889	0.889	0.931	0.915	0.870	0.796
PMut	0.800	0.556	0.769	0.600	0.667	0.611	0.407
SNPs3D	0.913	0.818	0.818	0.913	0.882	0.680	0.600
PANTHER	1.000	0.800	1.000	0.889	0.923	0.765	0.706
FATHMM-Weighted	0.900	0.696	0.842	0.795	0.811	1.000	0.811
FATHMM-Unweighted	0.857	0.459	0.895	0.375	0.569	1.000	0.569
MutationTaster	0.739	1.000	0.200	1.000	0.755	0.925	0.698
Condel	0.926	0.708	0.895	0.781	0.824	1.000	0.824
PROVEAN	1.000	0.900	1.000	0.917	0.952	0.778	0.741
Mutation assessor	1.000	0.500	1.000	0.304	0.590	0.422	0.426
MutPred	1.000	1.000	1.000	1.000	1.000	0.830	0.830
nsSNPAnalyzer	0.913	0.526	0.833	0.700	0.738	1.000	0.738
PhD-SNP	1.000	0.938	1.000	0.938	0.968	0.574	0.556
SNAP	0.955	0.846	0.917	0.913	0.914	0.648	0.593
SNPs&GO	1.000	0.708	1.000	0.563	0.788	0.660	0.520

PPV, positive predictive value; NPV, negative predictive value; PWeight, performance weight; WAccuracy, weighted accuracy.

1 See Materials and Methods section for calculations.

Statistical measures for the LGMD dataset can be seen in Table2, with accuracy values shown in Figure3B. Performance remained high for a few programs but greatly decreased in the majority of programs tested, demonstrating the reduced ability of prediction programs to correctly assign pathogenicity to recessive, loss-of-function mutations. The best overall performer in this dataset was again MutPred, with a weighted accuracy of 82.4%; this program also had the highest uncorrected accuracy (91.8%). Perfect specificity and PPV values were only achieved with one program (Mutation Assessor); however, this program also had the lowest sensitivity score (25.0%). MutationTaster had the only perfect sensitivity and NPV scores. The weakest overall performer with this dataset was PMut, with a weighted accuracy of 30.9%. FATHMM-Unweighted again had the lowest PPV score (59.3%). The lowest NPV (33.3%) was seen with nsSNPAnalyzer, while MutationTaster had the lowest specificity (45.0%).

**Table 2:** Statistical measures for program performance in the LGMD dataset

Program	PPV^1^	NPV^1^	Specificity^1^	Sensitivity^1^	Accuracy^1^	PWeight^1^	WAccuracy^1^
PolyPhen2-HumDiv	0.698	0.714	0.536	0.833	0.703	0.941	0.662
PolyPhen2-HumVar	0.700	0.704	0.679	0.724	0.702	0.838	0.588
SIFT	0.677	0.595	0.688	0.583	0.632	1.000	0.632
PMut	0.611	0.556	0.588	0.579	0.583	0.529	0.309
SNPs3D	0.947	0.500	0.875	0.720	0.758	0.805	0.610
PANTHER	0.778	0.609	0.778	0.609	0.683	0.719	0.491
FATHMM-Weighted	0.636	0.800	0.500	0.875	0.688	1.000	0.688
FATHMM-Unweighted	0.593	0.512	0.656	0.444	0.544	1.000	0.544
MutationTaster	0.738	1.000	0.450	1.000	0.784	0.750	0.588
Condel	0.722	0.688	0.688	0.722	0.706	1.000	0.706
PROVEAN	0.647	0.531	0.739	0.423	0.571	0.721	0.412
Mutation assessor	1.000	0.478	1.000	0.250	0.556	0.587	0.326
MutPred	0.912	0.926	0.893	0.939	0.918	0.897	0.824
nsSNPAnalyzer	0.923	0.333	0.667	0.750	0.737	1.000	0.737
PhD-SNP	0.762	0.700	0.737	0.727	0.732	0.574	0.420
SNAP	0.833	0.900	0.692	0.952	0.853	0.500	0.426
SNPs&GO	0.846	0.600	0.818	0.647	0.714	0.609	0.435

PPV, positive predictive value; NPV, negative predictive value; PWeight, performance weight; WAccuracy, weighted accuracy.

1 See Materials and Methods section for calculations.

### Top pathogenicity prediction programs

Regardless of the dataset used, a core group of pathogenicity prediction programs consistently performed near the top of the rankings. This group included MutPred, Condel, FATHMM-Weighted, SIFT, and nsSNPAnalyzer. As Condel includes the SIFT score as part of its algorithm, SIFT alone was removed from the core group. nsSNPAnalyzer was also removed, due to the large number of variants that could not be called. The performance of Condel, FATHMM-Weighted, and MutPred was reanalyzed, with an eye toward combined prediction power using multiple programs.

Variants were separated into the following categories: those with correct predictions from all three programs; those with incorrect predictions from all three programs; those with 2 correct predictions without a three-way agreement; and those with 2 incorrect predictions without a three-way agreement. The vast majority of variants (113 out of 121) could be placed into one of these categories (one variant was removed due to multiple missing outputs); the remaining number of variants either had three different predictions (Damaging, Possibly Damaging, and Benign) or two different predictions and one missing value.

**Figure 4:**
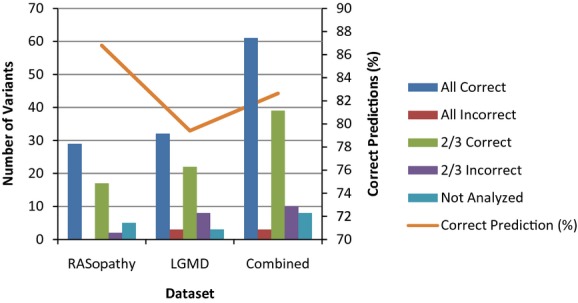
Performance of the combined program algorithm. The number of variants in each category (bars) and the percentage of correct predictions (line) are shown for each dataset when using the combined MutPred/Condel/FATHMM-Weighted method. RASopathy, *n* = 53; LGMD, *n* = 68; Combined, *n* = 121.

In the RASopathy dataset, the highest degree of concordance between top programs was seen between FATHMM-Weighted and Condel and between FATHMM-Weighted and MutPred. These combinations of programs had concordant predictions for 36 (67.7%) of 54 variants. The combined performance of FATHMM-Weighted, Condel, and MutPred was assessed on 53 variants (Fig.4); one variant was removed due to missing data for two of the three programs in question. All three programs correctly predicted the pathogenicity of 29 variants (18 credibly pathogenic and 11 credibly benign). An additional 17 variants (11 credibly pathogenic and 6 credibly benign) were correctly predicted by two of the three programs. No variants had three incorrect predictions, and only two variants had two incorrect predictions; both were credibly pathogenic mutations with two neutral calls. This amounted to 46 of 53 variants with trustworthy correct predictions, or 86.8% of the RASopathy variants.

In the LGMD dataset, all two-way combinations of programs produced the same results; concordant predictions were seen for 45 (66.2%) of 68 variants. When assessing the combined performance of FATHMM-Weighted, Condel, and MutPred (Fig.4), 32 variants were correctly predicted by all three programs (21 credibly pathogenic and 11 credibly benign). Two of the three programs correctly predicted the pathogenicity of 22 other variants (11 credibly pathogenic and 11 credibly benign). Unfortunately, three variants (two credibly pathogenic and one credibly benign) received incorrect predictions from all three programs, and an additional eight variants had two incorrect predictions (all credibly benign). In total, 54 of 68 variants received trustworthy correct predictions, or 79.4% of the LGMD variants. This was again a reflection of the decreased ability of prediction programs to accurately assess the pathogenicity of loss-of-function, recessive mutations.

When the results for the two separate datasets were combined, the accuracy of pathogenicity predictions was unchanged from the performance of the single best prediction program (Fig.4). Sixty-one variants, 39 credibly pathogenic and 22 credibly benign, were correctly predicted by all three programs. An additional 39 variants (22 credibly pathogenic and 17 credibly benign) were correctly predicted by two of the three programs. This amounted to 100 of 121 variants with correct predictions, or a prediction value of 82.6%.

Eight of the variants without correct predictions had inconsistent predictions using these three programs. Three variants had two different predictions in addition to a missing value (no prediction generated); all three of these variants were credibly pathogenic. Five variants (two credibly pathogenic and three credibly benign) had three different predictions. If we remove these variants from consideration, the correct prediction percentage increased to 88.5% (100 of 113). Thus, the use of a three-program system improved the accuracy of pathogenicity prediction over the use of any single program *only* when variants with relatively consistent predictions were analyzed. If all variants are included in the analysis, the combined approach was no better at predicting pathogenicity than the use of MutPred alone.

## Discussion

In this study, we compared the performance of multiple pathogenicity prediction programs on datasets that comprised variants selected for substantial evidence supporting their association with a particular disorder or their benign character. Previous comparative studies have utilized variant databases related to hereditary cancer syndromes, large all-encompassing datasets (HGMD, Swiss-Prot, etc.) or, rarely, congenital disease variants pulled from locus-specific databases (Capriotti et al. 2006; Bromberg and Rost 2007; Chan et al. 2007; Calabrese et al. 2009; Schwarz et al. 2010; Hicks et al. 2011; Thusberg et al. 2011; Choi et al. 2012; Shihab et al. 2013). To the best of our knowledge, this is the first analysis that specifically studied the performance of these programs on variants in Mendelian disorders unrelated to hereditary cancer with an emphasis on mode of inheritance and mechanism of disease.

The ability of prediction programs to differentiate between pathogenic and benign variants is based in part on the set of variants used in program training. The majority of programs were trained using large datasets of variants annotated in UniProt/SwissProt, HGMD, and/or dbSNP (Thomas et al. 2003; Ferrer-Costa et al. 2004; Bao et al. 2005; Capriotti et al. 2006; Yue et al. 2006; Bromberg and Rost 2007; Calabrese et al. 2009; Li et al. 2009; Adzhubei et al. 2010; Schwarz et al. 2010; Gonzalez-Perez and Lopez-Bigas 2011; Reva et al. 2011; Choi et al. 2012). However, these databases are not error-free, as previous studies have documented incorrect annotations in these resources (Tavtigian et al. 2008). Using incorrectly classified variants in training may increase the possibility of inaccurate calls from these programs. Other program generators have opted to use carefully curated databases of single gene or gene family variants in program training (Li et al. 2009; Gonzalez-Perez and Lopez-Bigas 2011; Reva et al. 2011; Choi et al. 2012). However, these are mainly variants associated with hereditary cancer syndromes. As these syndromes normally require multiple somatic mutation events after the initial germline mutation, these could constitute a very different mechanism of disease than that seen for variants involved in non-neoplastic constitutional disorders. Still others have utilized non-human variants, going as far phylogenetically as bacteriophages and viruses (Ferrer-Costa et al. 2004; Kumar et al. 2009; Choi et al. 2012). The differences in evolutionary influences on these diverse species could impact the prediction abilities of the programs that utilized these variant sets.

Given the wide range of training sets used to generate the predictive algorithms, one would expect that a program with greater overlap between training and testing sets would have better performance. However, we did not find any substantial correlation between the number of variants found both in our dataset and the original training set and overall accuracy of predictions. SNAP had the smallest amount of overlap in datasets; only two variants (both pathogenic RASopathy variants) from our total dataset were found in their training set. While SNAP was not among the top performers in this analysis, it was also not the weakest. In fact, SNAP had unweighted accuracy values of 85–91%; the main downfall of this program was the large number of variant predictions with low reliability scores.

On the opposite end of the spectrum, the program with the largest amount of overlap between training and testing datasets was FATHMM. Thirty-six (66.7%) of our RASopathy variants and 34 (50%) of our LGMD variants were also found in the training set for both the weighted and unweighted derivations of this program. FATHMM-Weighted was consistently one of the best programs when predicting the effect of our dataset variants. Interestingly, the vast majority of the overlapping variants were pathogenic in nature. While this fact is demonstrative when comparing specificity to sensitivity in the LGMD dataset (50.0% vs. 87.5%, respectively), these two values are very similar in the RASopathy dataset (84.2% and 79.4%). This again indicates the stronger predictive power of these algorithms with dominant, gain-of-function mutations.

The most accurate prediction program within the confines of our dataset was MutPred, with a weighted accuracy of 82.6% in the total dataset. Of note, the training set using for this algorithm fell within the middle of the pack in terms of amount of overlap with our dataset. Twenty-six (48.1%) RASopathy variants and 23 (33.8%) LGMD variants were found in both training and testing sets, for a combined overlap of 40.2%. This is the same amount of overlap seen with SIFT and SNPs&GO; however, the results are highly divergent. Therefore, the mere fact that identical variants can be found in both the training and testing set for a given predictive algorithm does not have significant bearing on its overall performance capabilities.

Several factors were concerning when analyzing the performance of these prediction programs using this highly curated dataset. First, five programs provided a reliability score with their pathogenicity prediction. While the addition of a reliability index would be helpful when analyzing novel variants, the sheer number of variants in our dataset with unreliable calls made these programs less useful. In this dataset of variants with an overwhelming preponderance of evidence toward a pathogenic or benign prediction, the presence of low-reliability predictions is unsettling. Second, half of the programs studied in this work produced predictions with “possible” pathogenicity. Again, when working with novel variants, a more quantitative approach to the relative pathogenicity of a change would be useful; however, when working with the credible variants in our dataset, it lent uncertainty to predictions that should be clear-cut.

Overall, we found that the majority of programs performed below their published accuracy levels using our defined dataset. While some drop in performance is to be expected, the decrease in accuracy was significant for a handful of programs. In particular, PMut originally stated a PPV of 83–87% (Ferrer-Costa et al. 2004); using our dataset, this program was the weakest performer, with a weighted accuracy of 35%. Similar results were seen for MutationAssessor; this program's stated accuracy was 79% (Reva et al. 2011), but it only achieved a weighted accuracy of 38% using our dataset of variants. For other programs, similar accuracies were seen as have been documented in other comparative studies (Capriotti et al. 2006; Bromberg and Rost 2007; Calabrese et al. 2009; Schwarz et al. 2010; Choi et al. 2012; Shihab et al. 2013). The exception to this rule was MutPred, the best performer in our dataset. The original publication describing this program stated the accuracy to be 62–80%, depending on the dataset used (Li et al. 2009). However, we achieved 83% accuracy, and similar results were seen in another comparative study (Shihab et al. 2013). The continued high performance of this pathogenicity prediction program across distinct variant datasets serves as confirmation that this program can be used reliably, regardless of the type of variant.

While some reports have debated the efficacy of including protein-based data along with multiple sequence alignments (Jordan et al. 2010), our results agree with the current consensus that consideration of protein features increases the performance of prediction programs (Tavtigian et al. 2008; Thusberg and Vihinen 2009). Our top three performers, MutPred, Condel, and nsSNPAnalyzer, all include protein features in their prediction algorithms. However, these results are tempered by the lack of protein data for a significant proportion of genes in our dataset. In particular, predictions could only be made for half of our dataset using nsSNPAnalyzer, given the lack of protein structures in the ASTRAL database. It should also be noted that, while we did not see any correlation between predictive accuracy and variant location within the protein structure, it is possible that this could play a key role in the prediction capability of some algorithms. As next-generation sequencing uncovers variants in poorly studied genes, this problem will only grow in effect.

In conclusion, the performance of pathogenicity prediction programs when analyzed using a dataset of clinically validated variants is mixed. Improvements in the quality of such predictions will be necessary to truly reduce the need for confirmatory functional testing, which remains outside the realm of clinical diagnostic laboratories. While these predictions should not be the final word in pathogenicity, they provide a useful way to filter the numerous variants that will be identified through clinical next-generation sequencing platforms.

## Author Contributions

L. C. W. S. participated in the design and coordination of the study, performed all analyses, and drafted the manuscript. S. H., D. L. T., S. R., J. M. G. F., and C. A. participated in the design and coordination of the study. R. E. P. conceived of the study, participated in its design and coordination, and helped to draft the manuscript. All authors read and approved the final manuscript.

## Conflict of Interest

None declared.

## Supporting Information

**Data S1.** Program Details

**Table S1.**RASopathy dataset variants: list of all variants in the RASopathy dataset.

**Table S2.**LGMD dataset variants: list of all variants in the LGMD dataset.

**Table S3.** Predictions for a selection of credibly pathogenic variants: predictions from selected credibly pathogenic variants from both the RASopathy dataset and the LGMD dataset.

**Table S4.** Variants with completely correct predictions: variants that were correctly predicted to be pathogenic or benign by all programs tested.
