# Supplementary material for: Variability in pathogenicity prediction programs: impact on clinical diagnostics
Source: Mol Genet Genomic Med. 2014 Dec 3;3(2):99–110. doi: 10.1002/mgg3.116 (PMC4367082; doi:10.1002/mgg3.116)
Supplement: Supplementary file 1 [file mgg30003-0099-sd1.doc]

**Supplementary Methods**

*Program Details*

**PolyPhen-2**

Website: http://genetics.bwh.harvard.edu/pph2/index.shtml

PolyPhen-2 utilizes eight sequence-based and three structure-based features in its prediction, which are selected by an iterative greedy algorithm. The algorithm output is then transformed to a prediction of impact by a naïve Bayes classifier. Separate outputs are produced for the HumDiv algorithm (trained using rare alleles causing Mendelian disease) and the HumVar algorithm (trained using more common alleles and nonsynonymous SNPs). Amino acid changes are classified as Probably Damaging, Possibly Damaging, or Benign.

**SIFT**

Website: http://sift.jcvi.org/www/SIFT_enst_submit.html

SIFT utilizes a multiple-sequence alignment generated using PSI-BLAST. The SIFT algorithm then determines probabilities for each amino acid at a given position, which are recorded in a scaled probability matrix. Amino acid changes are classified as Damaging if the scaled probability falls below a given threshold (0.05) or Benign. The SIFT algorithm also calculates a conservation value to ensure that each sequence alignment has optimum diversity. If the conservation value is above 3.25, the output is marked as low-confidence (classified as Possibly Damaging for this analysis).

**PMut**

Website: http://mmb2.pcb.ub.es:8080/PMut/

PMut utilizes both sequence-based (19) and structure-based (4) parameters in its prediction algorithm; structure parameters are only included in the model if such information is publicly available. The user-input version of this algorithm was used, rather than the Precalculated database. The output of the neural network is a number between 0 and 1, which is then transformed to a prediction of Pathological (values > 0.5) or Neutral (values < 0.5). In addition, a reliability index value (scale = 1-9) is calculated from the neural network output, such that values close to 0 or 1 will receive high reliability scores and values close to 0.5 will receive low reliability scores. For this analysis, only predictions with reliability scores ≥ 5 were used.

**SNPs3D**

Website: http://www.snps3d.org/

SNPs3D is a multi-module program designed to analyze the relationships between genes and disease. The SNP Analysis module Impact on Protein Structure and Function was used for amino acid change predictions. The sequence-based method was used for analysis, which utilizes a multi-sequence alignment to determine the extent and nature of amino acid conservation. The sequence alignment data is analyzed by a support vector machine (SVM) to assign a status to each amino acid change. The SVM output (a continuous numerical value) is transformed to a prediction of Deleterious (value ≤ -0.5) or Non-Deleterious (value ≥ 0.5). Values between these thresholds were classified as Possibly Deleterious for this analysis. When available, this module presents the user with different protein sequence options (designated by NCBI Protein Identifiers); whenever possible, the correct protein sequence was chosen for analysis. Output was only used if the correct wild-type amino acid was identified in the protein sequence used for comparison.

**PANTHER**

Website: http://www.pantherdb.org/tools/csnpScoreForm.jsp

PANTHER utilizes a native library of sequence alignments to generate a statistical model (Hidden Markov Model, HMM) regarding the conservation of amino acids at individual positions. These HMMs are used to score the functional likelihood of substitutions when analyzing amino acid changes. The HMM produces a “position-specific evoluntionary conservation” (PSEC) likelihood score for the given amino acid substitution (subPSEC), which ranges from 0 to -10. The HMM also produces a probability score regarding the pathogenicity of the substitution (P_deleterious_). The algorithm is set such that a subPSEC of -3 corresponds to a P_deleterious_ of 0.5. For this analysis, amino acid changes were classified in the following manner: Non-Deleterious if P_deleterious_ < 0.5, Possibly Deleterious if 0.5 ≤ P_deleterious_ ≤ 0.75, and Deleterious if P_deleterious_ > 0.75. If the amino acid change could not be aligned to the HMM, no output was produced.

**FATHMM**

Website: http://fathmm.biocompute.org.uk/inherited.html

FATHMM utilizes HMMs to generate a multiple-sequence alignment: one that is generated ab initio when searching public databases and curated HMMs from protein domain databases SUPERFAMILY and Pfam. These HMMs are used to interrogate the amino acid properties of the wild-type and substituted amino acid at a given position. Values corresponding to these properties are used in calculations to produce a predictive score. A loss in amino acid properties becomes a negative score and is translated as a deleterious change. This is the basis for their Unweighted/Species-Independent model. An additional model, Weighted/Species-Dependent, also includes a pathogenicity weight value in the final calculations. This pathogenicity weight is derived from the relative frequencies of disease-associated and neutral variants in a given protein domain. Both models were used for this analysis. FATHMM derived their own pathogenicity thresholds for the predictive score outputs; scores < -1.5 and -3.0 were deemed Deleterious for the Unweighted and Weighted models, respectively. Scores above these thresholds were deemed Non-Deleterious.

**MutationTaster**

Website: http://www.mutationtaster.org/

MutationTaster is the only program analyzed in this study which used base change as an input, rather than amino acid change. The MutationTaster algorithm, which is not well-described, combines information from public databases, including Ensembl, dbSNP, HapMap, and SwissProt. The algorithm output is analyzed by a naïve Bayes classifier, which generates a prediction of Disease-Causing or Polymorphism along with a p value for the reliability of the prediction. Only predictions with a p value ≥ 0.9 were used in this analysis.

**Condel**

Website: http://bg.upf.edu/condel/analysis

Condel combines the outputs of other protein prediction programs into a single score using a weighted average mechanism. The combined programs are PolyPhen-2, SIFT, MAPP, Logre, and Mutation Assessor. The weights are based on the individual scores from each program and each program’s individual ability to differentiate between truly deleterious and truly neutral mutations. The Condel score is a number between 0 and 1, with values < 0.5 called as Neutral and values > 0.5 called as Deleterious.

**PROVEAN**

Website: http://provean.jcvi.org/seq_submit.php

The PROVEAN algorithm first collects the components of a multiple-sequence alignment, then clusters them based on similarity. Each of the clusters is used to generate a Delta Score, which is computed by comparing the cluster sequence to the sequence with the amino acid change utilizing an amino acid substitution matrix. Each cluster-based Delta Score is then averaged to generate the final output score. More deleterious changes are associated with more negative outputs. PROVEAN utilizes a default cut-off value of -2.5 to determine pathogenicity. However, for this analysis, a more stringent cut-off of -4.1 was used, as detailed in the About section on the PROVEAN website. Thus, outputs were classified in the following manner: Benign if score ≥ -2.5; Possibly Damaging if -2.5 > score > -4.1; and Probably Damaging if score ≤ -4.1.

**Mutation Assessor**

Website: http://mutationassessor.org/

Mutation Assessor utilizes a multiple-sequence alignment to assess the conservation of an individual amino acid both within a large protein family and within smaller subfamilies. Both clusters are analyzed to determine evolutionary conservation, produced as conservation and specificity scores, using the distribution of amino acids, and therefore the implied entropy, at a given amino acid position. Larger scores indicate greater conservation i.e. less instances of an amino acid substitution at a given position, which suggests a greater risk of pathogenicity for substituted amino acids. The conservation and specificity scores are averaged to produce a Functional Impact Score (FIS). This score is then used to classify the pathogenicity of amino acid changes as neutral, low, medium, or high. For this analysis, neutral and low scores were classified as Benign, medium scores were classified as Possibly Damaging, and high scores were classified as Probably Damaging.

**MutPred**

Website: http://mutpred.mutdb.org/

MutPred bases its prediction algorithm on three categories of protein attributes: predicted protein structure and dynamics, predicted functional properties, and sequence-based evolutionary information. In total, 14 structural and functional properties were combined with SIFT and Pfam profile scores. All of the prediction classifiers were combined for analysis using a random forest method. Two types of output are generated: a general score (g) describing the probability that a given amino acid change is deleterious and individual p values for the functional impact of each protein property studied. The program is geared towards explaining the molecular mechanism of the pathogenicity of a given amino acid change; as such, different combinations of g scores and property p values are used to describe the confidence of hypotheses. However, this is beyond the scope of this analysis. Therefore, only g scores were used to describe the pathogenicity of a given amino acid change. For this analysis, amino acid changes were classified in the following manner: Non-Deleterious if g < 0.5, Possibly Deleterious if 0.5 ≤ g ≤ 0.75, and Deleterious if g > 0.75.

**nsSNPAnalyzer**

Website: http://snpanalyzer.uthsc.edu/

nsSNPAnalyzer combines analysis of a multiple-sequence alignment and protein structure analysis to determine the pathogenicity of a given amino acid change. Protein structural components are derived from homologous structures found in the ASTRAL database. SIFT scores are used to assay sequence conservation. The two types of data are combined to produce a prediction using a random forest method. The output of the nsSNPAnalyzer algorithm consists of a pathogenicity prediction (Disease or Neutral), along with the SIFT score and various protein structural features. If a homologous structure cannot be found in the ASTRAL database, no output is produced.

**PhD-SNP**

Website: http://snps.biofold.org/phd-snp/phd-snp.html

PhD-SNP utilizes two types of information when predicting pathogenicity. Both multiple-sequence alignment data and mutation environment within the surrounding protein sequence are used in separate SVM classifiers and then combined to produce a final output. The algorithm generates a prediction of either Disease or Neutral, along with a reliability index score (range = 0-9). For this analysis, only predictions with a reliability score ≥ 5 were used.

**SNAP**

Website: http://rostlab.org/services/snap/submit

SNAP utilizes both multiple-sequence alignment data as well as predicted protein structural components in its algorithm. The use of predicted protein information abolishes the need for a published structure. Each separate component of the algorithm was used as an input in a feed-forward neural network. The algorithm produces a predicted of Non-Neutral or Neutral for a given amino acid change, along with a reliability index score (range = 0-9) and accuracy rating (0-100%). For this analysis, only predictions with accuracy ratings ≥ 75% were used.

**SNPs&GO**

Website: http://snps-and-go.biocomp.unibo.it/snps-and-go/

SNPs&GO is an extension of PhD-SNP that incorporates additional evolutionary conservation data and gene ontology (GO) terms. Along with the multiple-sequence alignment and mutation environment data used in PhD-SNP, this SVM algorithm uses PANTHER output and a log-odds ratio score of the frequency of a given GO term between disease-causing and benign variants. Similar to PhD-SNP, the output consists of a pathogenicity prediction of Disease or Neutral, along with a reliability index score (range = 0-9). Only predictions with reliability scores ≥ 5 were used in this analysis.
