# Supplementary material for: Variability in pathogenicity prediction programs: impact on clinical diagnostics
Source: Mol Genet Genomic Med. 2014 Dec 3;3(2):99–110. doi: 10.1002/mgg3.116 (PMC4367082; doi:10.1002/mgg3.116)
Supplement: Supplementary file 2 [file mgg30003-0099-sd2.doc]

Supplementary Table 1. RASopathy Dataset Variants.

| Gene (Nucleotide ID)  [OMIM ID] | Credibly Pathogenic Variants | HGMD Accession ID | HGMD Variant Class | Credibly Benign Variants | dbSNP ID | dbSNP Validation Method^a^ |
| --- | --- | --- | --- | --- | --- | --- |
| *PTPN11* | c.172A>G (p.N58D) | CM044250 | DM^b^ | c.565T>G (p.S189A) | rs79068130 | 1 |
| (NM_002834.3) | c.181G>A (p.D61N) | CM021127 | DM | c.879C>G (p.H293Q) | rs117730996 | 2,3 |
| [176876] | c.182A>G (p.D61G) | CM013415 | DM | c.1124A>G (p.Y375C) | rs41299183 | 1,3 |
|  | c.188A>G (p.Y63C) | CM013416 | DM |  |  |  |
|  | c.215C>G (p.A72G) | CM013417 | DM |  |  |  |
|  | c.236A>G (p.Q79R) | CM013420 | DM |  |  |  |
|  | c.417G>C (p.E139D) | CM021132 | DM |  |  |  |
|  | c.836A>G (p.Y279C) | CM021133 | DM |  |  |  |
|  | c.844A>G (p.I282V) | CM013421 | DM |  |  |  |
|  | c.853T>C (p.F285L) | CM024733 | DM |  |  |  |
|  | c.854T>C (p.F285S) | CM021134 | DM |  |  |  |
|  | c.922A>G (p.N308D) | CM013422 | DM |  |  |  |
|  | c.923A>G (p.N308S) | CM021135 | DM |  |  |  |
|  | c.1403C>T (p.T468M) | CM021672 | DM |  |  |  |
|  | c.1493G>T (p.R498L) | CM041071 | DM |  |  |  |
|  | c.1507G>A (p.G503R) | CM060440 | DM |  |  |  |
|  | c.1510A>G (p.M504V) | CM013423 | DM |  |  |  |
|  | c.1529A>G (p.Q510R) | CM052358 | DM |  |  |  |
| *SOS1* | c.322G>A (p.E108K) | CM070271 | DM | c.73C>T (p.P25S) | rs139592595 | 1,2 |
| (NM_005633.3) | c.806T>G (p.M269R) | CM070275 | DM | c.280A>G (p.I94V) | rs144757941 | 1,2 |
| [182530] | c.1294T>A (p.W432R) | CM070287^c^ | DM | c.553A>G (p.I185V) | rs143962515 | 1,2,3 |
|  | c.1297G>A (p.E433K) | CM070281 | DM | c.929G>A (p.R310H) | rs143481916 | 1,2 |
|  | c.1644T>G (p.S548R) | CM105345 | DM | c.1705C>G (p.L569V) | rs200786705 | 1 |
|  | c.1654A>G (p.R552G) | CM070269 | DM | c.1964C>T (p.P655L) | rs56219475 | 1,2,3 |
|  | c.1655G>T (p.R552M) | CM116030 | DM | c.2122G>A (p.A708T) | rs140811086 | 1,2,3 |
|  |  |  |  | c.2371C>A (p.L791I) | rs142004123 | 1,2,3 |
|  |  |  |  | c.3032A>G (p.N1011S) | rs8192671 | 1,2,3,4 |
| *HRAS* (NM_176795.3) [190020] | c.34G>A (p.G12S) | CM053283 | DM |  |  |  |
| *KRAS* (NM_033360.2) [190070] | c.64A>G (p.Q22R) | CM070966^d^ | DM |  |  |  |
| *NRAS* (NM_002524.4) [164790] | c.35G>T (p.G12V) | N/A^e^ | N/A |  |  |  |
| *BRAF* (NM_004333.4) | c.770A>G (p.Q257R) | CM060874 | DM |  |  |  |
| [164757] | c.1455G>C (p.L485F) | CM060880 | DM |  |  |  |
| *RAF1* (NM_002880.3) | c.770C>T (p.S257L) | CM073301 | DM | c.122G>A (p.R41Q) | rs145611571 | 1,2 |
| [164760] | c.1472C>T (p.T491I) | CM073291 | DM | c.923C>T (p.P308L) | rs5746220 | 1,2,3, 4 |
| *MAP2K1/MEK1* (NM_002755.3) | c.199G>A (p.D67N) | CM076269 | DM | c.848C>T (p.A283V) | rs144080051 | 1,2 |
| [176872] |  |  |  | c.1139G>A (p.G380D) | rs28730804 | 1,2,4 |
| *MAP2K2/MEK2* (NM_030662.3) | c.400T>C (p.Y134H) | CM080434 | DM | c.844C>T (p.P282S) | rs142307980 | 1,2 |
| [601263] |  |  |  | c.1162C>T (p.R388W) | rs144383241 | 1,2,3 |
| *SHOC2* (NM_007373.3) [602775] | c.4A>G (p.S2G) | CM095445 | DM | c.1594A>G (p.S532G) | rs145463534 | 1,2,3 |

^a^1:Multiple independent submissions, 2:Frequency data, 3:1000 Genomes, 4:HapMap Project; ^b^Disease-Causing Mutation; ^c^c.1294T>C (identical amino acid change) in HGMD; ^d^c.65A>G (identical amino acid change) in HGMD; ^e^Variant not found in HGMD but described as Pathogenic in ClinVar (rs121913237)
