# Supplementary material for: Variability in pathogenicity prediction programs: impact on clinical diagnostics
Source: Mol Genet Genomic Med. 2014 Dec 3;3(2):99–110. doi: 10.1002/mgg3.116 (PMC4367082; doi:10.1002/mgg3.116)
Supplement: Supplementary file 3 [file mgg30003-0099-sd3.doc]

Supplementary Table 2. LGMD Dataset Variants.

| Gene (Nucleotide ID)  [OMIM ID] | Credibly Pathogenic Variants | HGMD Accession ID | HGMD Variant Class | Credibly Benign Variants | dbSNP ID | dbSNP Validation Method^a^ |
| --- | --- | --- | --- | --- | --- | --- |
| *SGCA* | c.100C>T (p.R34C) | CM971332 | DM^b^ | c.421C>A (p.R141S) | rs35130237 | 1,3 |
| (NM_000023.2) | c.229C>T (p.R77C) | CM951152 | DM | c.1120C>T (p.R374C) | rs35495899 | 1,2,3 |
| [600119] | c.371T>C (p.I124T) | CM971339 | DM |  |  |  |
|  | c.409G>A (p.E137K) | CM971340 | DM |  |  |  |
|  | c.739G>A (p.V247M) | CM951157 | DM |  |  |  |
|  | c.850C>T (p.R284C) | CM971346 | DM |  |  |  |
| *SGCB* | c.31C>G (p.Q11E) | CM971347 | DM | c.794C>T (p.T265I) | rs116214830 | 1,2,3 |
| (NM_000232.4) | c.341C>T (p.S114F) | CM971349 | DM |  |  |  |
| [600900] | c.355A>T (p.I119F) | CM983768 | DM |  |  |  |
|  | c.452C>G (p.T151R) | CM951158 | DM |  |  |  |
| *SGCD* (NM_000337.5) [601411] |  |  |  | c.290G>A (p.R97Q) | rs45559835 | 1,2,3 |
| *SGCG* | c.787G>A (p.E263K) | HM040058 | DM | c.8G>A (p.R3H) | rs35105771 | 1,2,3 |
| (NM_000231.2) |  |  |  | c.347G>A (p.R116H) | rs17314986 | 1,2,3,4 |
| [608896] |  |  |  | c.539A>T (p.E180V) | rs114160429 | 1,2,3 |
| *CAPN3* | c.145C>T (p.R49C) | CM076055 | DM | c.73C>T (p.H25Y) | rs61735534 | 1,2,3 |
| (NM_000070.2) | c.245C>T (p.P82L) | CM022771 | DM | c.551C>T (p.T184M) | rs35889956 | 1,2,3 |
| [114240] | c.319G>A (p.E107K) | CM053159 | DM | c.706G>A (p.A236T) | rs1801449 | 1,2,3,4 |
|  | c.479C>G (p.A160G) | CM041734 | DM |  |  |  |
|  | c.649G>A (p.E217K) | CM990305 | DM |  |  |  |
|  | c.956C>T (p.P319L) | CM970213 | DM |  |  |  |
|  | c.1063C>T (p.R355W) | CM051869 | DM |  |  |  |
|  | c.1303G>A (p.E435K) | CM041739 | DM |  |  |  |
|  | c.1319G>A (p.R440Q) | CM041740 | DM |  |  |  |
|  | c.1342C>T (p.R448C) | CM990313 | DM |  |  |  |
|  | c.1468C>T (p.R490W) | CM950194 | DM |  |  |  |
|  | c.1714C>T (p.R572W) | CM970221 | DM |  |  |  |
|  | c.2243G>A (p.R748Q) | CM970224 | DM |  |  |  |
|  | c.2306G>A (p.R769Q) | CM950197 | DM |  |  |  |
|  | c.2338G>C (p.D780H) | CM050543 | DM |  |  |  |
|  | c.2393C>A (p.A798E) | CM076058 | DM |  |  |  |
|  |  |  |  |  |  |  |
|  |  |  |  |  |  |  |
|  |  |  |  |  |  |  |
|  |  |  |  |  |  |  |
|  |  |  |  |  |  |  |
| *DYSF* | c.898G>C (p.G300R) | CM055148^c^ | DM | c.386G>A (p.G129E) | rs34997054 | 1,2,3 |
| (NM_001130983.1) | c.1123G>A (p.V375L) | CM052859^c^ | DM?^d^ | c.568C>G (p.L190V) | rs13407355 | 1,2,3,4 |
| [603009] | c.3044A>G (p.Y1015C) | CM103813^c^ | DM | c.1052C>T (p.A351V) | rs115279465 | 1,2,3 |
|  | c.3118C>T (p.R1040W) | N/A^e^ | N/A | c.1372G>A (p.E458K) | rs61740288 | 1,2,3 |
|  | c.3895A>G (p.I1299V) | CM980575^c^ | DM | c.2056C>G (p.L686V) | rs74423119 | 1,2,3 |
|  | c.3995G>T (p.R1332L) | CM103814^c^ | DM? | c.2503A>G (p.I835V) | rs34671418 | 1,2,3 |
|  |  |  |  | c.2591A>C (p.D864A) | rs35884879 | 1,2,3 |
|  |  |  |  | c.2951A>C (p.K984T) | rs34061568 | 1,2 |
|  |  |  |  | c.3068G>A (p.R1023Q) | rs34211915 | 1,2,3 |
|  |  |  |  | c.3217G>C (p.A1073P) | rs34660230 | 1,2 |
|  |  |  |  | c.3290G>A (p.R1097H) | rs59915619 | 1,2,3 |
|  |  |  |  | c.3355G>A (p.G1119S) | rs202000264 | 3 |
|  |  |  |  | c.3728G>A (p.R1243H) | rs2303603 | 1,2,3,4 |
|  |  |  |  | c.3763C>T (p.R1255W) | rs146970014 | 1,2,3 |
|  |  |  |  | c.3917C>A (p.T1306K) | rs116426399 | 1,2,3 |
|  |  |  |  | c.5692G>A (p.D1898N) | rs115013641 | 1,2,3 |
| *FKRP* | c.266C>T (p.P89L) | CM062722 | DM | c.822C>G (p.I274M) | rs77138370 | 1,3 |
| (NM_024301.4) | c.826C>A (p.L276I) | CM013805 | DM |  |  |  |
| [606596] | c.898G>A (p.V300M) | CM033474 | DM |  |  |  |
| *ANO5* |  |  |  | c.259G>A (p.V87I) | rs34994927 | 1,2,3 |
| (NM_213599.2) |  |  |  | c.604G>A (p.E202K) | rs115750596 | 1,2,3 |
| [608662] |  |  |  | c.616A>G (p.T206A) | rs78266558 | 1,2,3 |
|  |  |  |  | c.966A>T (p.L322F) | rs7481951 | 1,2,3,4 |
|  |  |  |  | c.2387C>T (p.S796L) | rs61910685 | 1,2,3 |

^a^1:Multiple independent submissions, 2:Frequency data, 3:1000 Genomes, 4:HapMap Project; ^b^Disease-Causing Mutation; ^c^Different isoform used in HGMD, corresponds to -3 bp and -1 amino acid; ^d^Possibly Disease-Causing Mutation; ^e^Variant not found in HGMD but described as Pathogenic in Leiden Muscular Dystrophy Database (different isoform, corresponds to -3 bp and -1 amino acid)
