# Supplementary material for: Variability in pathogenicity prediction programs: impact on clinical diagnostics
Source: Mol Genet Genomic Med. 2014 Dec 3;3(2):99–110. doi: 10.1002/mgg3.116 (PMC4367082; doi:10.1002/mgg3.116)
Supplement: Supplementary file 4 [file mgg30003-0099-sd4.doc]

**Supplementary Table 3A. Predictions for a Selection of Credibly Pathogenic RASopathy Variants**.

| Prediction Program | *PTPN11*  c.188A>G  (p.Y63C) | *SOS1*  c.1294T>A (p.W432R) | *PTPN11*  c.844A>G  (p.I282V) | *SHOC2*  c.4A>G  (p.S2G) |
| --- | --- | --- | --- | --- |
| PolyPhen2-HumDiv | Probably Damaging | Probably Damaging | Benign | Possibly Damaging |
| PolyPhen2-HumVar | Probably Damaging | Probably Damaging | Benign | Possibly Damaging |
| SIFT | Damaging | Damaging | Tolerated | *Damaging |
| PMut | Neutral | Pathological | Neutral | Neutral |
| SNPs3D | Deleterious | Deleterious | Non-Deleterious | N/A |
| PANTHER | Deleterious | *Deleterious | Deleterious | N/A |
| FATHMM-Weighted | Damaging | Damaging | Damaging | Tolerated |
| FATHMM-Unweighted | Damaging | Damaging | Tolerated | Tolerated |
| MutationTaster | Disease-Causing | Disease-Causing | Disease-Causing | Disease-Causing |
| Condel | Deleterious | Deleterious | Neutral | Deleterious |
| PROVEAN | Probably Damaging | Probably Damaging | Probably Damaging | Neutral |
| Mutation Assessor | Probably Damaging | Possibly Damaging | Tolerated | Tolerated |
| MutPred | Deleterious | Deleterious | Deleterious | Possibly Damaging |
| nsSNPAnalyzer | Disease | Disease | Neutral | N/A |
| PhD-SNP | Disease | Disease | Neutral | Neutral |
| SNAP | Disease | Disease | Neutral | Disease |
| SNPs&GO | Disease | Disease | Neutral | Neutral |

**Supplementary Table 3B. Predictions for a Selection of Credibly Pathogenic LGMD Variants.**

| Prediction Program | *SGCA*  c.229C>T  (p.R77C) | *CAPN3*  c.1714C>T (p.R572W) | *SGCB*  c.31C>G  (p.Q11E) | *DYSF*  c.3895A>G (p.I1299V) |
| --- | --- | --- | --- | --- |
| PolyPhen2-HumDiv | Probably Damaging | Probably Damaging | Benign | Benign |
| PolyPhen2-HumVar | Probably Damaging | Probably Damaging | Benign | Benign |
| SIFT | Damaging | Damaging | Tolerated | Tolerated |
| PMut | Pathological | Pathological | Neutral | Neutral |
| SNPs3D | Deleterious | Deleterious | Non-Deleterious | N/A |
| PANTHER | Deleterious | Deleterious | Non-Deleterious | N/A |
| FATHMM-Weighted | Damaging | Damaging | N/A | Tolerated |
| FATHMM-Unweighted | Damaging | Damaging | Tolerated | Tolerated |
| MutationTaster | Disease-Causing | Disease-Causing | *Polymorphism | *Disease-Causing |
| Condel | Deleterious | Deleterious | Neutral | Neutral |
| PROVEAN | Probably Damaging | Probably Damaging | Neutral | Neutral |
| Mutation Assessor | Possibly Damaging | Probably Damaging | Tolerated | N/A |
| MutPred | Deleterious | Deleterious | Possibly Damaging | Benign |
| nsSNPAnalyzer | N/A | Disease | N/A | N/A |
| PhD-SNP | Disease | Disease | Neutral | Neutral |
| SNAP | Disease | Disease | Disease | Neutral |
| SNPs&GO | Disease | Disease | Neutral | N/A |

Two credibly pathogenic variants from the RASopathy (**A**) and LGMD (**B**) datasets with highly correlative predictions and two credibly pathogenic variants with weakly correlative predictions are shown with predictive outputs from each program. Color system: Red=Pathogenic; Yellow=Possibly Pathogenic; Green=Benign; Light Gray=Correct prediction with low confidence; Dark Gray=Incorrect prediction with low confidence; White=No prediction generated.
