# Supplementary material for: Variability in pathogenicity prediction programs: impact on clinical diagnostics
Source: Mol Genet Genomic Med. 2014 Dec 3;3(2):99–110. doi: 10.1002/mgg3.116 (PMC4367082; doi:10.1002/mgg3.116)
Supplement: Supplementary file 5 [file mgg30003-0099-sd5.doc]

**Supplementary Table 4. Variants with Completely Correct Predictions**.

| Gene | Variant | Pathogenicity | # Correct Calls | # Unreliable Calls^a^ | # Missing Calls^b^ |
| --- | --- | --- | --- | --- | --- |
| *PTPN11* | c.188A>G (p.Y63C) | Credibly Pathogenic | 16 | 1 | 0 |
|  | c.1493G>T (p.R498L) | Credibly Pathogenic | 16 | 0 | 1 |
|  | c.879C>G (p.H293Q) | Credibly Benign | 16 | 1 | 0 |
| *SOS1* | c.553A>G (p.I185V) | Credibly Benign | 16 | 0 | 1 |
| *MAP2K1* | c.1139G>A (p.G380D) | Credibly Benign | 12 | 3 | 2 |
| *CAPN3* | c.1342C>T (p.R448C) | Credibly Pathogenic | 17 | 0 | 0 |
|  | c.1468C>T (p.R490W) | Credibly Pathogenic | 16 | 1 | 0 |
|  | c.1714C>T (p.R572W) | Credibly Pathogenic | 17 | 0 | 0 |
| *DYSF* | c.898G>C (p.G300R) | Credibly Pathogenic | 11 | 1 | 5 |
|  | c.568C>G (p.L190V) | Credibly Benign | 11 | 1 | 5 |
|  | c.2503A>G (p.I835V) | Credibly Benign | 11 | 2 | 4 |
| *ANO5* | c.2387C>T (p.S796L) | Credibly Benign | 12 | 2 | 3 |

^a^Low-confidence predictions; ^b^Program generated no prediction (N/A)
